# Using Systematic Reviews and Meta-Analyses to Support Regulatory Decision Making for Neurotoxicants: Lessons Learned from a Case Study of PCBs

**DOI:** 10.1289/ehp.0901835

**Published:** 2010-02-22

**Authors:** Michael Goodman, Katherine Squibb, Eric Youngstrom, Laura Gutermuth Anthony, Lauren Kenworthy, Paul H. Lipkin, Donald R. Mattison, Judy S. LaKind

**Affiliations:** 1 Department of Epidemiology, Emory University School of Public Health, Atlanta, Georgia, USA; 2 Department of Medicine, University of Maryland School of Medicine, Baltimore, Maryland, USA; 3 Department of Psychology and; 4 Department of Psychiatry, University of North Carolina at Chapel Hill, Chapel Hill, North Carolina, USA; 5 Center for Autism Spectrum Disorders, Children’s National Medical Center, Washington, DC, USA; 6 Department of Pediatrics; 7 Department of Psychiatry and; 8 Department of Neurology, George Washington University School of Medicine, Washington, DC, USA; 9 Center for Development and Learning, Kennedy Krieger Institute, Baltimore, MD, USA; 10 Department of Pediatrics, Johns Hopkins University School of Medicine, Baltimore, Maryland, USA; 11 *Eunice Kennedy Shriver* National Institute of Child Health and Human Development, National Institutes of Health, Department of Health and Human Services, Bethesda, Maryland, USA; 12 LaKind Associates, LLC, Catonsville, Maryland, USA; 13 Department of Epidemiology and Preventive Medicine, University of Maryland School of Medicine, Baltimore, Maryland, USA; 14 Department of Pediatrics, Milton S. Hershey Medical Center, Penn State College of Medicine, Hershey, Pennsylvania, USA

**Keywords:** domain, function testing, meta-analysis, neurodevelopment, neurotoxicants, PCBs, risk assessment, weight of evidence

## Abstract

**Background:**

Epidemiologic weight-of-evidence reviews to support regulatory decision making regarding the association between environmental chemical exposures and neurodevelopmental outcomes in children are often complicated by lack of consistency across studies.

**Objective:**

We examined prospective cohort studies evaluating the relation between prenatal and neonatal exposure to polychlorinated biphenyls (PCBs) and neurodevelopment in children to assess the feasibility of conducting a meta-analysis to support decision making.

**Data extraction/synthesis:**

We described studies in terms of exposure and end point categorization, statistical analysis, and reporting of results. We used this evaluation to assess the feasibility of grouping studies into reasonably uniform categories.

**Results:**

The current literature includes 11 cohorts of children for whom effects from prenatal or neonatal PCB exposures were assessed. The most consistently used tests included Brazelton’s Neonatal Behavioral Assessment Scale, the neurologic optimality score in the neonatal period, the Bayley Scales of Infant Development at 5–8 months of age, and the McCarthy Scales of Children’s Abilities in 5-year-olds. Despite administering the same tests at similar ages, the studies were too dissimilar to allow a meaningful quantitative examination of outcomes across cohorts.

**Conclusions:**

These analyses indicate that our ability to conduct weight-of-evidence assessments of the epidemiologic literature on neurotoxicants may be limited, even in the presence of multiple studies, if the available study methods, data analysis, and reporting lack comparability. Our findings add support to previous calls for establishing consensus standards for the conduct, analysis, and reporting of epidemiologic studies in general, and for those evaluating the effects of potential neurotoxic exposures in particular.

Extensive literature exists on the use and interpretation of neurodevelopmental tests that serve as outcome measures in population studies examining effects of environmental exposures. However, conclusions about the presence or absence of a causal relation between an exposure to a specific toxicant and a particular outcome are generally based on weight of evidence (WOE), because even well-designed studies are subject to methodologic limitations that are unavoidable in observational research; no single study can be considered sufficient for producing definitive results. For this reason, it is crucial that the scientific and regulatory communities are able to evaluate findings across studies before rendering the WOE-based conclusions. The term “WOE” has several possible definitions; we refer to WOE as a methodology with a “simple premise: that all available evidence should be examined and interpreted” (Weed 2005). It is important to clarify that for the purposes of this review we focus on weight of epidemiologic (vs., e.g., toxicologic) evidence. Such WOE evaluation is possible only if the studies under review use the same or similar methods of exposure assessment, outcome ascertainment, data analysis, and reporting of results.

To provide a methodologic framework for a review of the association between *in utero* and early-life exposures to environmental chemicals and neurodevelopmental outcomes in children, we considered epidemiologic studies that focused on prenatal and neonatal exposure to polychlorinated biphenyls (PCBs). Our selection of PCBs as the exemplar chemical class was based on two main considerations. First, it was important to select an environmental chemical or chemical class for which a sufficient body of peer-reviewed literature was available for evaluation. Scientific studies on PCBs and neurodevelopment date back to the early 1980s and include cohorts from several countries. Second, for the purposes of this examination of neurodevelopmental epidemiologic studies and implications for interstudy comparison, we sought to select a chemical or chemical class for which substantial uncertainty exists regarding presence or absence of a causal relation between prenatal/neonatal exposure and neurodevelopmental outcomes. With respect to PCBs, recent reviews appear to indicate considerable disagreement among experts (Boucher et al. 2009; Cicchetti et al. 2004; Kimbrough and Krouskas 2003; Schantz et al. 2003), and controversy exists as to whether PCBs at current environmental levels of exposure are in fact neurotoxicants (Winneke et al. 2003). Although others have provided reviews of the PCB neurodevelopment literature (Boucher et al. 2009; Kimbrough and Krouskas 2003), their value for WOE is weakened by differing and sometimes idiosyncratic matching of neurodevelopmental assessment instruments to putative neurodevelopmental domains and by a lack of formal assessment of consistency across studies addressing the same exposure–outcome associations for the same or similar study populations.

The purpose of this review is not to weigh in on the ongoing debate over neurodevelopmental effects of PCBs. Instead, we used studies of PCBs as a vehicle for evaluating the state of the science in population research aimed at investigating the relation between prenatal or neonatal exposures to environmental chemicals and performance on neurodevelopmental function tests. In this review, we present results from our assessment of the epidemiologic literature on the relation between PCBs and neurodevelopment regarding *a*) the consistency of study methods with respect to exposure assessment, outcome ascertainment, and data analysis; and *b*) the feasibility of conducting a quantitative WOE assessment of existing epidemiologic data (i.e., a meta-analysis). The goals are to develop a general framework for assessing the body of evidence in neurodevelopmental environmental epidemiology studies and to offer recommendations to guide future research such that results will be more amenable to WOE reviews in support of regulatory decision making.

## Methods

### Identification/selection of studies

We used several electronic data sources [PubMed (http://www.ncbi.nlm.nih.gov/pubmed), Cochrane Library (http://www.thecochranelibrary.com), EMBASE (http://www.embase.com/home), PsycINFO (http://www.apa.org/pubs/databases/psycinfo/index.aspx), and Web-of-Knowledge (http://apps.isiknowledge.com)] to conduct the initial literature search, with an end date of December 2009. Using keywords “polychlorinated,” “biphenyls,” “PCB,” “PCBs,” “children,” “prenatal,” “neurodevelopmental,” and “neurobehavioral,” as well as various combinations of these keywords, we selected relevant articles that investigated the neurodevelopmental effects of environmental PCB exposures in children (poisoning events were not considered). We reviewed secondary references of retrieved articles to identify publications not captured by the electronic search. We conducted additional literature searches to identify relevant reports and textbook chapters that were not published in the peer-reviewed literature.

The prospective longitudinal design provides the most informative data for examining outcomes associated with *in utero* and early-life exposures (Amler et al. 2006; Wigle et al. 2008). For this reason, our search of the literature focused specifically on cohort studies that recruited participants either prenatally or soon after birth and linked various measures of pre- and postnatal PCB exposures to neurodevelopmental outcomes at different ages; at the time of this review, some of the studies had conducted only one neurodevelopmental evaluation.

### Literature review

We retrieved and reviewed the publications identified via the literature search (~ 60 articles) and extracted information on each relevant study with respect to its methods of data collection, analysis and reporting. Extracted information was categorized according to the following characteristics: *a*) cohort description—year of enrollment, geographic location, and ages at which neurodevelopmental/neurobehavioral tests were administered; *b*) exposure categorization—whether information was based on maternal dietary questionnaires or measured (e.g., in breast milk, maternal serum, or cord blood) and units of measures [e.g., nanograms per gram, parts per billion, or toxic equivalents (TEQs)]; *c*) tests used to define the end points of interest—neurologic [e.g., neurologic optimality scores (NOSs)], cognitive [e.g., Bayley Scales of Infant Development (BSID)], or other tests assessing specific domains of functioning; and *d*) analysis and reporting of result—linear regression coefficients with and without log transformation of variables, parametric or nonparametric comparisons of outcomes in two or more groups, or qualitative description of results.

This characterization of the cohort studies allowed us to search for reasonably homogeneous groups of articles that could then be included in a systematic analysis. Within each group, we assessed the feasibility of a meta-analysis of the published data. It is a common practice that a minimum data set needed for the systematic analysis should include at least three similar studies, in which measures of effect and corresponding measures of variance for the same exposure–outcome association within the same age group either were reported by the study authors or could be calculated using the data from the original articles (Treadwell et al. 2006).

## Results

### Overview of PCB cohort studies

**Figure 1:**
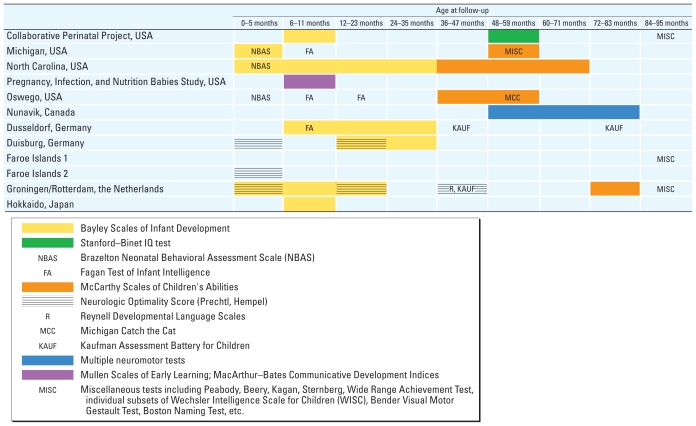
Summary of tests used in the PCB cohorts by age (for children up to 7 years of age at follow-up). Collaborative Perinatal Project, USA (Daniels et al. 2003); Michigan, USA (Jacobson et al. 1984); North Carolina, USA (Rogan et al. 1986); Pregnancy, Infection, and Nutrition Babies Study, USA, (Pan et al. 2009); Oswego, USA (Lonky et al. 1996); Nunavik, Canada (Després et al. 2005); Dusseldorf, Germany (Winneke et al. 1998); Duisburg, Germany (Wilhelm et al. 2008); Faroe Islands 1 (Grandjean et al. 2001); Faroe Islands 2 (Steuerwald et al. 2000); Groningen/Rotterdam, the Netherlands (Huisman et al. 1995a); Hokkaido, Japan (Nakajima et al. 2006). Additional testing was conducted with the Oswego cohort at 8 and 9 years of age, the Netherlands cohort at 9 years of age, and the Michigan cohort at 11 years of age.

The current published literature includes 11 cohort studies of children for whom pre- or neonatal PCB exposures were measured (as maternal blood levels during pregnancy, cord blood, breast milk concentrations, or combinations of these) or estimated. These studies represent a wide range of populations (in terms of geography and year of enrollment) recruited either at birth or prenatally, some of which were followed for several years (up to 11 years for one cohort). Geographically, five of the cohorts were recruited in the United States and Canada, five in Europe, and one in Japan. The neurodevelopmental outcomes at various ages were described in at least 40 different articles with publication dates spanning a 26-year interval from 1984 through 2009. Figure 1 summarizes tests administered in each cohort study at different ages through the seventh year of life. [Figure 1 also includes a twelfth cohort—the Pregnancy, Infection, and Nutrition Babies Study (Pan et al. 2009)—that has reported results for only one neurodevelopmental function testing period to date and used function tests that differed from those used for the other cohort studies. This cohort is not discussed further.] Most (9 of 11) cohorts were evaluated for neurologic and behavioral function or cognitive ability during the first year of life. After the first year of age, the frequency of testing decreased. Importantly, although not shown in Figure 1, after 8 years of age the available neurodevelopmental data become even more sparse.

### Feasibility of quantitative analysis

Our review of each cohort summarized in Figure 1 showed that the opportunities for a WOE review and/or meta-analysis of studies that used the same tests among children of the same or similar age appear to be most promising in the first and the fifth years of life. As noted in “Methods,” our goal was to identify reasonably homogeneous groups of at least three studies. Studies were considered eligible for a meta-analysis if *a*) similar tests were administered at similar ages, *b*) exposure was measured and reported in comparable ways, *c*) results represented comparable measures of effect, and *d*) for the purposes of weighting in a meta-analysis, measures of effect were accompanied by corresponding measures of variance.

The earliest opportunity to assess consistency of findings across studies in terms of participants’ age was presented in the neonatal period (i.e., within 28 days postpartum). Studies conducted in the United States and in Europe used different types of testing to examine neurobehavioral function in newborns. As shown in Table 1, the three U.S. studies included the Michigan cohort (Jacobson et al. 1984), the Oswego cohort (Lonky et al. 1996; Stewart et al. 2000), and the North Carolina cohort (Rogan et al. 1986). All of the U.S. studies administered Brazelton’s Neonatal Behavioral Assessment Scale (NBAS), which was divided into seven clusters. Six of those clusters—response decrement, orientation, tonicity, range of state, regulation of state, and autonomic maturity—are considered behavioral. One cluster—reflex—is aimed at evaluating neurologic function.

The Michigan and Oswego cohorts were given the NBAS test within the first 3 days of life. Both studies carried out multivariate analyses to link fish consumption (as a surrogate for exposure to PCBs) to NBAS score; however, the outcome definitions for the two cohorts differed from each other. Specifically, the Michigan study (Jacobson et al. 1984) used a single NBAS result obtained on the third day of life, whereas the Oswego study (Lonky et al. 1996) defined the outcome as the difference between two assessments conducted in the second and the first day after birth. Further, the multivariate analyses in the two studies [linear regression for the Michigan study and multivariate analysis of covariance (MANCOVA) for the Oswego study] produced results that could not be compared and/or combined quantitatively. A second publication based on the Oswego cohort (Stewart et al. 2000) examined the association between NBAS and cord blood PCBs in addition to fish consumption, which was already assessed by Lonky et al. (1996). The exposure was assessed using four metrics (total PCBs, lightly chlorinated PCBs, moderately chlorinated PCBs, and highly chlorinated PCBs), and the outcomes in this study were assessed separately at each time interval (1 day and 2 days of life) both as the NBAS score for each cluster and as an overall proportion of poor scores. The data were analyzed using a test for trend statistic; however, the quantitative results were reported only for the second day of life assessment and only for highly chlorinated PCBs.

The third study (the North Carolina cohort) that administered NBAS did so between the first and third week of life (Rogan et al. 1986). The analytic approach (linear regression) used in the North Carolina study was similar to that of the Michigan study (Jacobson et al. 1984), but the exposure measures differed (PCBs measured in breast milk were compared with estimates of PCB exposure based on fish consumption information or cord blood levels). In addition, the results were presented in terms of *p-*values without reporting the regression coefficients. Thus, despite the consistent use of NBAS in the first week of life by these three cohorts, differences in methods for estimating exposures and in reporting of outcomes preclude conducting a quantitative systematic review across the cohorts. It is worth noting that even if the statistical method had been consistent across studies, the differences in choice of covariates would still have rendered it very difficult to synthesize the effect sizes across studies (Lipsey 2001).

The three European studies of neonatal outcomes (Table 2) were conducted in Duisburg, Germany (Wilhelm et al. 2008), the Netherlands (Huisman et al. 1995a), and the Faroe Islands (Steuerwald et al. 2000). All three studies used the NOS, a combined measure that consists of 60 components with an optimal range of results predefined for each item, with the final score calculated as the total number of optimal items (Touwen et al. 1980).

All three of these studies conducted the NOS assessment between 1 and 3 weeks of life and in that respect are comparable to the North Carolina cohort. Two of the three European studies—the Duisburg (Wilhelm et al. 2008) and Faroe Islands (Steuerwald et al. 2000) cohorts—performed linear regression analyses to examine the relation of NOS scores at 2 weeks of life to PCB levels in both milk and maternal blood samples; however, quantitative results are given only for the Duisburg cohort (Wilhelm et al. 2008). In addition, different analytes were selected for exposure assessment in these two studies. Wilhelm et al. (2008) examined PCBs together with polychlorinated dibenzo-*p*-dioxins and dibenzofurans (PCDD/Fs), whereas Steuerwald et al. (2000) expressed exposure as ∑PCB (the sum of PCB congeners). the Netherlands cohort (Huisman et al. 1995a) dichotomized the NOS using the median PCB concentration in the study population as the cutoff. For the resulting binary outcome in the logistic regression analyses, the independent variable of interest was the log-transformed ∑PCB and various ∑PCB subsets (e.g., planar versus nonplanar). Again, despite the availability of three studies using the same neurodevelopmental test, differences in methods for estimating exposures and in reporting of outcomes preclude conducting a quantitative systematic review.

Six cohort studies used the same test—BSID—to assess the cognitive function of their participants between 5 and 8 months of age and thus could provide comparable data (Table 3). Three of these studies were conducted in the United States. The Michigan and the North Carolina cohort studies (Gladen et al. 1988; Jacobson et al. 1986) have been discussed previously in the context of neonatal assessment. The third U.S. study (Daniels et al. 2003) represents a multicenter effort—called the Collaborative Perinatal Project—that recruited participants from several sites (Baltimore, MD; Boston, MA; Buffalo, NY; Memphis, TN; Minneapolis, MN; New Orleans, LA; New York, NY; Philadelphia, PA; Portland, OR; Providence, RI; Richmond, VA). Among the three European studies shown in Table 3, two were carried out using the same cohort in Dusseldorf, Germany (Walkowiak et al. 2001; Winneke et al. 1998), and one was conducted using a subset of the previously discussed cohort of children from the Netherlands (Koopman-Esseboom et al. 1996). One additional study in this category was performed with a cohort of children from Sapporo, Japan (the Hokkaido Study on Environment and Children’s Health) (Nakajima et al. 2006).

The versions of the BSID assessment used in these studies included two main scores: the Mental Development Index (MDI) and the Psychomotor Development Index (PDI). As shown in Table 3, all studies evaluating the relation between BSID in the first year of life and PCB exposure used linear regression to estimate the effect. However, the reporting and interpretation of the linear regression coefficients differed across the studies. Although we identified four studies that examined the relationship between MDI and PCB concentrations in maternal or cord blood, the results in these studies represented different measures of effect. In two of the four studies, the regression coefficients represented change in MDI per unit of PCB increase (micrograms per liter or nanograms per gram) (Daniels et al. 2003; Winneke et al. 1998); in two other studies (Koopman-Esseboom et al. 1996; Nakajima et al. 2006), the corresponding coefficients represented change in MDI per natural logarithm of exposure. Similarly, among the three studies that reported the association between MDI and PCBs in breast milk, only two (Gladen et al. 1988; Winneke et al. 1998) reported their findings as comparable regression coefficients per 1 ppm or 1 ng/g of exposure; the third study (Jacobson et al. 1986) simply noted a lack of association. Another publication that evaluated the relation between breast milk PCB levels and MDI (Walkowiak et al. 2001) used the same German cohort data as used by Winneke et al. (1998) but reported linear regression coefficients per logarithm base 2 of exposure. The results of studies for PDI at 5–8 months of life provided even less comparable information. Among the seven publications (based on six different cohort studies), only five calculated and reported regression coefficients, and only four of those studies were based on independent data. As was the case with MDI, it was not possible to identify three independent studies that could be combined in a meta-analysis because of the variability of exposure characterization and/or methods of expressing study results. Overall, even with the use of the Bayley Scales across several cohorts at similar times in life, a quantitative systematic review across cohorts was not possible.

Three cohort studies (Michigan, Oswego, and Dusseldorf) evaluated their participants at 6–7 months of age using the Fagan Test of Infant Intelligence. Although all three studies measured PCB cord blood concentrations (among other metrics), the specific congeners were different. Moreover, the association between exposure and outcome was assessed using different statistical methods: multiple linear regression that used PCB levels as a continuous variable in two studies (Jacobson et al. 1985 and Winneke et al. 1998) and an *F*-test for trend that used a four-level cord blood PCB categorization in the third study (Darvill et al. 2000). All three analyses appear to have controlled for different sets of confounders. Thus, the studies that administered the Fagan test were as heterogeneous as the studies that used BSID at roughly the same age.

The only remaining opportunity to assess the feasibility of conducting a meta-analysis was in a group of studies assessing cognitive function during the fifth year of life. As shown in Figure 1, three cohort studies in the United States (Michigan, Oswego, and North Carolina) evaluated the cognitive function of their participants between the fourth and the fifth birthdays using McCarthy Scales of Children’s Abilities and were considered as candidates for inclusion in a meta-analysis (Gladen and Rogan 1991; Jacobson et al. 1990; Stewart et al. 2003). All three of these studies reported the results for the General Cognitive Index (GCI) of the McCarthy Scales. Only one of these studies also presented the results separately for the Verbal, Quantitative, Perceptual–Performance Memory, and Motor Scales (Jacobson et al. 1990).

Table 4 summarizes results for the three studies evaluating the association between perinatal PCB exposure and GCI. It is evident that despite testing the same hypothesis, the differences across the three studies were too pronounced to allow meaningful conclusions about the presence or absence of consistency in findings. Specifically, although the Michigan study (Jacobson et al. 1990) conducted linear regression analyses for cord blood and breast milk PCB exposures, only cord blood results were provided in their publication. The North Carolina study (Gladen and Rogan 1991) used breast milk concentrations to estimate exposure, but the data were analyzed using ANCOVA procedures, and the quantitative results were not reported. The Oswego cohort study (Stewart et al. 2003) was similar to the Michigan study in that they both estimated exposure based on PCB concentration in cord blood. However, unlike the Michigan study, the Oswego findings were presented not as regression coefficients but as linear *F*-test results, which divided exposure into four ordinal categories. As with our other attempts at a systematic review across cohorts, there was insufficient consistency with exposure measures and outcome reporting to conduct such a review.

## Discussion

Despite the relatively large body of literature on potential associations between early-life exposure to PCBs and adverse neurodevelopmental effects, controversy still exists over whether PCBs are in fact neurotoxicants, and to date, the U.S. Environmental Protection Agency has not established regulatory guidance values for PCBs based on neurotoxicity. Such regulatory decision making generally relies on a WOE assessment of studies, which in turn requires comparability across studies. Unfortunately, our examination of the PCB neurodevelopmental epidemiology literature found a lack of interstudy consistency. Even for age intervals examined by several research groups, presumably testing the same hypothesis, a meta-analysis of PCB studies is not possible at this time. Moreover, the frequency of evaluations decreased substantially and the data became increasingly sparse as the cohorts became older. This likely presents a missed research opportunity because testing in older children may be more reliable, and perhaps more informative with respect to the long-term prognosis (Sattler 2008).

As noted above, it is not the purpose of this review to weigh in on the ongoing debate over neurodevelopmental effects of PCBs, but rather to use the PCB neurodevelopmental epidemiology literature as the basis for describing generalizable issues related to interstudy consistency. Replication of findings, often referred to as “repeating a study,” is a crucial aspect of the scientific method. Ability to repeat or reproduce a result leads to generalizable inferences, rather than merely to isolated and uncertain findings (Lindsay and Ehrenberg 1993). In the field of medical research, there is consensus that replication (or other substantiation) of clinical trials is a requirement for approval of drugs and medical devices (Berlin and Colditz 1999). Unlike testing of drugs and devices, most data generated by environmental research involving human subjects are observational in nature, and thus the conditions within a study are far less controlled. As noted in a recent review (Bellinger 2009), researchers conducting observational studies have great latitude in how exposure and outcome are measured and expressed, which methods for examining associations are employed, and which analyses among the myriad typically conducted are reported. In this regard, the epidemiologic studies that rely on neurodevelopmental function test results as the end points of interest may be particularly affected by variability of study methods and reporting. This is attributable to the large number of available test batteries, each of which can offer different combinations of subtests (Youngstrom et al. 2010). Even within subtests, there are different scales and cutoff points for categorizing responses. If these are used and reported selectively, it can be very difficult to determine whether two different studies have demonstrated similar or conflicting results or have assessed overlapping but slightly different functions (Garabrant 2000).

Although consistency in study methods and reporting is a critical prerequisite of any WOE review, it is important to stress that consistency of methods alone is not sufficient for drawing conclusions about causation. By combining several studies, meta-analyses have an inherent ability to detect relatively small statistically significant departures from null. However, these relatively precise meta-estimates may not accurately reflect the true association unless the analyses take into consideration potential sources of systematic error that may affect reviews of the literature. One source of error that warrants consideration in any systematic review is publication bias, which can occur because studies with statistically significant positive findings are more likely to be published than are studies with null results. Publication bias has been shown to be of particular importance in observational studies (Easterbrook et al. 1991). Another closely related concept is selective reporting bias within published studies and is defined as “selection on the basis of the results of a subset of the original variables recorded for inclusion in a publication” (Dwan et al. 2008). Consider, for instance, the Netherlands cohort study that administered neurologic testing and calculated the NOS at two different ages: 10–21 days and 18 months of age (Huisman et al. 1995a, 1995b). Although the two follow-ups tested the same hypothesis, the two statistical analyses were markedly different: logistic regression at 10–21 days and linear regression at 18 months of age. Perhaps more important, the strongest inverse association (between nonplanar PCBs and NOS) observed in newborns does not seem to have been reexamined (or at least not reported) in the 18-month-olds.

The search for sources of error in any systematic review inevitably leads to evaluation of individual study quality. Issues that need to be addressed usually include magnitude of nonparticipation or loss to follow-up, misclassification of exposure and/or outcome, and ability to control for extraneous factors, all of which may introduce bias. For example, an important method of minimizing information bias is making sure that persons administering the test (and at a later age perhaps also subjects themselves) are unaware of the participants’ exposure status. Among studies summarized in this review (Tables 1–4), many indicated that they implemented blinding; however, in two instances (Gladen et al. 1988; Huisman et al. 1995a) the investigators were unaware of the results of laboratory analyses but knew which children were breast-fed; this information was used in estimating PCB exposure. In addition, several studies did not mention blinding procedures in their respective methods sections (Daniels et al. 2003; Gladen and Rogan 1991; Jacobson et al. 1986; Stewart et al. 2003; Wilhelm et al. 2008).

In the absence of comparable published information, one potential method for assessing the consistency of findings across studies would be to obtain the original data and then either compare the results using the same statistical methods or combine the data in a pooled analysis. For example, pooling of the data might be helpful in bringing together the three studies (Koopman-Esseboom et al. 1996; Daniels et al. 2003; Nakajima et al. 2006) that examined the association between prenatal maternal blood levels of PCBs and BSID scores but focused on different sets of congeners, used different modeling approaches, and controlled for different covariates. Such pooled analyses would be possible for only some of the many associations examined to date and would, of course, require the cooperation of researchers and depend on their willingness to share data. Perhaps more important, future studies of chemical exposures and neurodevelopmental outcomes must build on previous research with the aim of facilitating WOE assessments. Repeated calls for establishing consensus standards for the conduct, analysis, and reporting of epidemiologic studies have been voiced in a variety of areas of research, including those related to the effects of neurotoxicant exposures (Bellinger 2009).

WOE assessment is essential to interpreting results of epidemiology studies of neurodevelopment and chemical exposure. Yet even for chemicals that have been studied for their neurotoxicity for decades, there is still controversy over whether WOE is sufficient to state unequivocally that they are neurotoxicants, or to define the dose–response relationship. We used PCBs as a case study to highlight the need for improved inter- and intrastudy consistency in the selection of neurodevelopment function tests and domains to be evaluated, exposure assessment, and/or method of analyzing/reporting data.

## Conclusions

We conclude with the following recommendations: First, although novel approaches for assessing neurodevelopment will continue to be developed and should be used, it is important that future research include measures comparable to those used by past researchers. The lack of inclusion of comparable measures will hinder our ability to conduct WOE assessments. We recommend that key individuals and international organizations determine and establish the specific comparable measures that should be included in each study. This is not intended to be a prescriptive list that would limit future investigators’ novel approaches, but rather a methodologic feature that would permit future evaluations by scientists and regulators.

Similarly, future investigators will likely have new tools (or a favored tool) for assessing exposure to environmental chemicals. These include traditional exposure assessments, biomonitoring, and use of biomarkers of exposure. A standard, baseline metric of exposure should be derived that is evaluated as a minimum exposure metric for all studies (other types of exposure assessments could be conducted in addition to this baseline metric) to again allow for interstudy comparisons.

Third, although efforts are being made within certain agencies (e.g., the National Institutes of Health) to require sharing of raw data, a broader effort is needed to ensure that study data are available for WOE assessments. This will not occur without in-place requirements (i.e., agency-required data sharing) as part of research-funding mechanisms.

In addition, selection of statistical methods for analyzing data from complex data sets has been the subject of intense and sometimes acrimonious debate (Kimbrough and Krouskas 2003). To this end, we recommend that an expert panel composed of statisticians, neurologists, psychologists, psychometricians, epidemiologists, and exposure and risk assessors from academia and government who have not been part of past environmental neurodevelopmental epidemiology studies (and can therefore bring fresh perspectives) be convened to discuss and recommend best practices.

Last, journals could facilitate progress by either accepting or requiring the archival of tables of summary statistics, such as unadjusted correlations, means, and standard deviations, perhaps augmented by a description of patterns of missing data. Some publication manuals, style guides, and other guidelines recommend the archiving of sufficient descriptive statistics to allow independent analyses of the data (American Psychological Association 2010). Techniques are available that would allow the inclusion of these summary tables in subsequent meta-analyses (Becker 1996), and they also would establish a “least common denominator” of data reporting that would still represent an advance over the current fragmented and hard to synthesize state of the literature.

We recognize that reaching agreement within the scientific community on the recommendations above will be difficult. However, we believe that without some consensus on each of these issues, our ability to truly evaluate neurodevelopmental risks associated with chemical exposures will not be possible.

## Figures and Tables

**Table 1:** U.S. studies of neurologic and behavioral function among newborns using the Brazelton NBAS^a^ in relation to measures of prenatal/early-life PCB exposure.

Reference, cohort	Age at test administration	Exposure measure	Tests measuring association	Results
Jacobson et al. 1984, Michigan, USA	3 days	Fish consumption as surrogate for PCB exposure	Chi-square test where outcome is dichotomized as normal vs. worrisome and exposure is defined as none vs. high	Chi-square test with level of significance (e.g., < 0.1, < 0.05)
			Partial correlations adjusted (one at a time) for birth weight, head circumference, gestational age, and BEFM	Partial correlations (with level of significance, but without variance) reported for lability of states (a subset of RaS), RaS, AM, and R; other clusters not reported
			Multiple linear regression, adjusted for birth weight, head circumference, gestational age	Regression coefficients (with level of significance, but without variance) reported for lability of states, AM and R; other clusters not reported
		Cord serum PCBs (congeners not specified)	Linear *F*-test adjusted for birth weight, head circumference, gestational age, and BEFM	For cord blood, text indicates no significant association

Rogan et al. 1986, North Carolina, USA	1–3 weeks	PCB in breast milk fat (congeners not specified)	Multiple linear regression, adjusted (simultaneously) for maternal age, education, occupation, smoking, drinking, fish intake, and anesthesia; infant’s race, sex, birth weight, jaundice, age of testing, interval between testing and feeding	Results reported as significant for T and R (regression coefficients not reported); results for other clusters not reported, but text notes significant associations were found only for T and R and that for RD *p*-value was 0.07

Lonky et al. 1996, Oswego, New York, USA^b^	1 day and 2 days	Fish consumption as surrogate for PCB exposure	MANCOVA with principal components analysis; outcome: the difference between two assessments; exposure: three fish consumption groups (high, low, and none)	*F*-values with levels of significance (Bonferroni adjusted) reported for six clusters, all except RD; direction/magnitude of difference reported for R, AM, and RD across three levels of fish consumption (but without variance)

Stewart et al. 2000, Oswego, New York, USA	1 day and 2 days	∑PCB (69 congeners) in cord blood; lightly, moderately, and highly chlorinated PCB congeners in cord blood; fish consumption as surrogate for PCB exposure	Linear *F*-test for each age (1 day and 2 days) analyzed separately across four categories of highly chlorinated PCBs (nondetectable, low, medium, and high) adjusted for unspecified covariates and three fish consumption groups (high, low, and none); linear *F*-test across exposure categories where outcome is defined as percentage of poor NBAS scores	Results for total ∑PCB and lightly and moderately chlorinated PCBs not reported; results for highly chlorinated PCBs at 1 day noted as showing no differences; results for highly chlorinated PCB at 2 days given as linear term (*F*-statistic) for R, AM, and RD with either *p*-values or levels of significance; results for percentage of poor NBAS scores given as *F*-test and a *p*-value for highly chlorinated cord blood PCB and fish consumption; text indicates that lightly and moderately chlorinated PCB were not related to percentage of poor NBAS scores

BEFM, Ballard Examination for Fetal Maturity.

a Brazelton NBAS clusters: response decrement (RD), orientation, tonicity (T), range of state (RaS), regulation of state (ReS), autonomic maturity (AM), and reflex (R).

b NBAS seven clusters administered twice: at 12–25 and 25–48 hr of life.

**Table 2:** European studies that examined neurologic and behavioral function among newborns using the NOS in relation to measures of prenatal or early-life PCB exposure.

Reference, cohort	Age at test administration	Exposure measure	Tests measuring association	Results
Huisman et al. 1995a, the Netherlands	10–21 days	Individual, ∑, and TEQs: planar PCBs (3 congeners) and nonplanar PCBs (up to 23 congeners) in breast milk fat; individual and ∑PCBs (4 congeners) in maternal plasma and cord plasma	Logistic regression with outcome dichotomized as optimal vs. nonoptimal and results expressed as odds ratios for doubling of exposure adjusted for maternal age, study center, alcohol use	Reported as odds ratios and corresponding 95% confidence intervals
Steuerwald et al. 2000, Faroe Islands	2 weeks	∑PCBs (three congeners) in breast milk, lipid-adjusted ∑PCBs (3 congeners) in maternal serum	Linear regression where exposure and outcome were expressed as continuous variables controlling for Hg and other (not specified) covariates	Text indicates weak positive association; regression results not reported; Spearman’s correlation coefficients reported (but without variance)
Wilhelm et al. 2008, Duisburg, Germany	2 weeks	PCDD/F + PCB TEQs (congeners not specified) in maternal blood and breast milk lipid	Linear regression presented as MD, where MD – 1 = β for doubling of exposure adjusted for maternal age, gestational age, birth weight, alcohol intake, age of examination, maternal Pb and Hg exposure, and examiner	Reported as linear regression coefficients and corresponding 95% confidence intervals

Abbreviations: Hg, mercury; MD, mean differences; Pb, lead.

**Table 3:** Studies using MDI and PDI (BSID) at 5–8 months of age in relation to measures of prenatal or early-life PCB exposure.

Reference, cohort	Age at test administration	Exposure measure	Tests measuring association	Results
Jacobson et al. 1986, Michigan, USA	5 months	Fish consumption as surrogate for PCB exposure; cord blood PCBs (congeners not specified); breast milk PCBs (congeners not specified)	Linear regression and ANCOVA; details not reported	Text indicates that no measures of exposure were related to MDI or PDI; quantitative results not provided
Gladen et al. 1988, North Carolina, USA	6 months	PCB in breast milk fat (congeners not specified) at birth used as measure of transplacental exposure; estimated cumulative exposure to PCB in breast milk (from birth to the age of test)	Multiple linear regression per 1 ppm, adjusted for maternal age, race, education, occupation, smoking, drinking, infant’s sex, gestational age, birth weight, head circumference, jaundice, duration of breast-feeding, number of older siblings, abnormal reflexes on NBAS age of testing, and center/examiner	Both MDI and PDI results reported as regression coefficients, standard errors, and two-sided *p*-values
Koopman-Esseboom et al. 1996, the Netherlands	7 months	Prenatal exposure: ∑PCBs (24 congeners) in maternal blood drawn in the last month of pregnancy; postnatal exposure: ∑PCBs (4 congeners and 24 congeners) in breast milk	Multiple linear regression expressed per ln(ng/g) adjusted for gestational age, parity, HOME score, and duration of breast-feeding	Result for MDI reported as regression coefficients, standard errors, and two-sided *p*-values; results for PDI not reported
Winneke et al. 1998,^a^ Dusseldorf, Germany	7 months	∑PCBs (three congeners) in cord blood and breast milk	Multiple linear regression expressed per ng/g adjusted for maternal age, education, vocabulary, smoking and drinking, duration of breast-feeding, birth weight, HOME score, Apgar score, cord blood lead level, and neonatal illness	Both MDI and PDI results for both cord blood and breast milk PCB levels reported as regression coefficients, standard errors, and one-sided *p*-values
Walkowiak et al. 2001,^a^ Dusseldorf, Germany	7 months	∑PCBs (three congeners) in breast milk and cord serum	Multiple linear regression per log_2_(ng/g) adjusted for parity, smoking, and body mass index	For breast milk PCBs, both MDI and PDI results reported as regression coefficients, standard errors, and one-sided *p*-values; for cord blood PCBs, text indicates that associations were small or positive; quantitative results not provided
Daniels et al. 2003, Collaborative Perinatal Project, USA	8 months	∑PCBs (11 congeners) in maternal blood drawn in the third trimester	Multiple linear regression per μg/L adjusted for research center, maternal education, triglycerides, cholesterol, and birth order of the child	Both MDI and PDI results reported as regression coefficients, standard errors, and two-sided *p*-values
Nakajima et al. 2006, Hokkaido, Japan	6 months	∑PCBs (total 14 congeners and by different categories of congeners) and TEQs in maternal blood drawn in the third trimester of pregnancy or after delivery	Multiple linear regression per ln(ng/g) or per TEQ adjusted for gestational age, smoking and caffeine intake during pregnancy, and blood-sampling time	Both MDI and PDI results reported as regression coefficients and two-sided *p*-values

HOME, Home Observation for Measurement of the Environment.

a Two studies based on the same cohort; Winneke et al. (1998) reported BSID results at 7 months of age only, whereas Walkowiak et al. (2001) evaluated the same subjects again at 18 and 30 months of age and reported results at all three ages.

**Table 4:** Studies using the GCI of the McCarthy Scales of Children’s Abilities in the fifth year of age in relation to measures of prenatal or early-life PCB exposure.

Reference, cohort	Age at test administration	Exposure measure	Tests measuring association	Results
Jacobson et al. 1990, Michigan, USA	4 years	Cord blood PCBs (congeners not specified)	Multiple linear regression per log*_x_*(ng/mL) +1 adjusted for maternal age, gravidity, and examiner^a^	PCB result reported as a regression coefficient with a two-sided *p*-value
		Breast milk PCBs (congeners not specified)	Details of linear regression modeling for breast milk exposure not reported	PCB regression; results not provided

Gladen and Rogan 1991, North Carolina, USA	4–5 years	PCB (congeners not specified) in breast milk fat at birth used as a measure of transplacental exposure; estimated cumulative exposure to PCB in breast milk (from birth to the age of test)	ANCOVA by dividing transplacental exposure into eight categories and cumulative breast milk exposure into five categories, adjusted for maternal age, race, education, occupation, smoking, drinking, infant’s sex, number of older siblings, feeding pattern	For transplacental PCB exposure text indicates no association; quantitative results not provided; for cumulative breast milk exposure text indicates some variation but the pattern “did not suggest cause and effect”; quantitative results not provided

Stewart et al. 2003, Oswego, New York, USA	4.5 years	∑PCBs in cord blood (17 congeners)	Linear *F*-test across four PCB exposure categories (nondetectable, low, medium, and high) adjusted for various combinations of (not specified) covariates	Result reported as a linear term without a measure of variance (all *p*-values marked as nonsignificant)

a Details of log transformation are not given.
